# Pulmonary Consumption in Man and Animals

**Published:** 1843-05

**Authors:** 


*Pulmonary Consumption in Man and Animals.*—Rayer read a long memoir on this subject to the French Academy of Sciences, on the 25th July last. The following are his conclusions:—

1*st.* Of all chronic diseases, pulmonary consumption is the most common in man and animals.

2*d.* In man and other mammalia, the tuberculous matter can always be easily distinguished from recent pus, which is loaded with seed globules. In birds, the characters of tuberculous matter are less marked; foreign bodies introduced into the lungs and flesh, do not produce an opaque white humor, full of seed globules, but a dry yellowish matter, without globules, whose physical characters approach those of tubercles in the mammalia. In reptiles, fishes, and insects, the characters of tubercle are still less distinct.

3*d.* Pus, in the mammalia, and chiefly in the horse, if it remains long in any organ, undergoes successive transformations, so that at last it resembles tuberculous matter.

4*th.* Pulmonary tubercles in man and in the quadrumana have generally a gray color; in the *pommelière* of the cow the tuberculous matter is yellowish.

5*th.* In man and animals the softening of the tuberculous matter in the centre cannot be attributed to inflammation, as no pus globules are ever seen; the softening, however, on the circumference is much assisted by the inflammation of the contiguous tissues, and it is almost always mixed with pus globules.

6*th*. The yellow matter which is found in the cysts of hydatids in the ruminantia after the destruction or spontaneous rupture of the cysts, has some analogy with tubercular matter, (*pommelière;*) but the cysts of this yellow matter almost always contain the debris of the hydatid pouch, and sometimes a certain quantity of pus.

7*th*. The earthy or calcareous concretions (principally composed of carbonate and phosphate of lime) which are met with in the lungs of man, and other animals, ought not to be considered, as they are even to this day, as being almost always a modification of tubercle. They are frequently in man, and very often in the horse, the residue of a small depot of pus

8*th*. Granulations depending on worms and on glanders form in the lungs of several animals, which ought to be distinguished from tuberculous granulations.

9*th*. Among the quadrumana, and some birds brought from warm climates, consumption shows itself in its greatest degree, and almost to the exclusion of other chronic diseases. It is equally often produced in other animals coming from the north, as the rein-deer, by a change of climate and food.

10*th*. Consumption, which is rare among the domestic herbivorous (solipedes) animals, is still rarer among the carnivorous. However, notwithstanding the preservative influence of a strong constitution, and an animal diet, several of the carnivora, as the domestic cat, and especially the lion, tiger, and jaguar brought to our country, have been seized with phthisis. This rarity of consumption is also seen in birds which are rapacious.

11*th*. By a kind of contradiction, the domestic dog among the carnivora, and the hare among the herbivora, are less subject to tubercles than to cancer, a disease which Camper thought never attacked the lower animals.

12*th*. Among the ruminantia, and especially in the class boves, consumption is often associated with vesicular worms, and particularly with the echinococcus; but, contrary to the opinion several times given, there is no transformation or succession between these hydatids and tubercles.

13*th*. Fatty liver is the common accompaniment of consumption in man, and general obesity in birds.

14*th*. The alterations of bone which are observed in simiæ affected with this disease, and more particularly in those from

New Holland, appear analogous to the deformities, swelling, and spongy softening of the bones seen in phthisical and scrofulous children. Similar alterations of bone take place among the carnivora brought to this country from a warmer climate.

15*th*. If the frequency of pneumonia, and the rarity of consumption in the domestic dog, would appear to do away with any connection between these diseases, the same thing cannot be said to occur in the calf, cow, or female ass, as in these the deposit of tuberculous matter almost always coincides with a chronic and increasing pneumonia.

16*th*. Consumption is hereditary, but it is almost never congenital, even in the rudimentary state.

17*th*. Among people affected with phthisis, the semen contained in the seminal vesicles contains few or no spermatic animalcules.

18*th*. Ulcers in the larynx, trachea, and bronchi, do not indicate the same thing in man and animals. In the former, they are almost always owing to consumption, and sometimes to syphilis; among the quadrumana to general tubercular diathesis, and among quadrupeds almost always to glanders.

19*th*. In pneumo-thorax, mouldy vegetations may form on the altered pleura of a phthisical patient, as sometimes takes place in the air-sacs of birds affected with consumption, or other diseases of the respiratory organs. In this case, as in all those which have been observed among the vertebrata, the development of these parasites is a secondary phenomenon.

Rayer classes the causes of consumption under four heads: A domestic state and captivity for the inferior animals, and misery and fatigue for man; and he concludes that science, which is altogether powerless in curing the disease, ought not to be so in preventing it.—*Lond. and Edin. Monthly Journ. Med. Sci.*, Dec. 1842.

In opposition to the seventh conclusion of R., Prus asserts that for ten years he had been endeavoring to ascertain, at the Bicêtre and the Salpetrière, the curability of tubercles, that he has been led more and more to believe that the earthy concretions, which are almost always at the summit of the lung, are only modified tubercles; that they are generally accompanied by traces of cicatrization; that sometimes old cavities, lined with a new mucous membrane, co-exist along with them; and, lastly, that it is easy to find, even in the same lung, tubercles in different degrees of progress, which present all the phases of this secretion, from its origin to its earthy state, and frequently the same cyst contains both tubercle and earthy matter. Such are the reasons which make him dissent from Rayer's theory, that the earthy concretions are the result of a depôt of purulent matter in the substance of the lung, and not a modification of tubercle.—*Ibid*, February, 1843.—*Amer. Journ. Med. Sci.*

